# *In Vitro* Conservation of Sweet Potato Genotypes

**DOI:** 10.1155/2014/208506

**Published:** 2014-01-19

**Authors:** Maria de Fátima Arrigoni-Blank, Fernanda Ferreira Tavares, Arie Fitzgerald Blank, Maria Clézia dos Santos, Thays Saynara Alves Menezes, Aléa Dayane Dantas de Santana

**Affiliations:** Department of Agronomic Engineering, Federal University of Sergipe, Avenida Marechal Rondon S/N, São Cristóvão, Brazil

## Abstract

The aim of this study was to develop a protocol for the *in vitro* conservation of sweet potato genotypes using the slow growth technique. The first experiment was conducted in a 4 × 5 × 2 factorial scheme, testing four genotypes (IPB-007, IPB-052, IPB-072, and IPB-137), five concentrations of abscisic acid (ABA) (0.0, 1.0, 2.0, 4.0, and 8.0 mg*·*L^−1^), and two temperatures (18 and 25°C). The second experiment was conducted in a 4 × 3 × 3 factorial scheme at 18°C, testing four genotypes (IPB-007, IPB-052, IPB-072, and IPB-137), three variations of MS salts (50, 75, and 100%), and three concentrations of sucrose (10, 20, and 30 g*·*L^−1^). Every three months, we evaluated the survival (%), shoot height, and shoot viability. *In vitro* conservation of the sweet potato genotypes IPB-052 and IPB-007 was obtained over three and six months, respectively, using MS medium plus 2.0 mg*·*L^−1^ of ABA at either 18 or 25°C. Genotypes IPB-072 and IPB-137 can be kept for three and six months, respectively, in MS medium without ABA at 18°C. It is possible to store IPB-052 and IPB-072 for six months and IPB-007 and IPB-137 for nine months using 30 g*·*L^−1^ of sucrose and 50% MS salts.

## 1. Introduction

The sweet potato [*Ipomoea batatas* (L.) Lam] is a rustic tropical vegetable crop, which is well suited for cultivation in various regions of Brazil. Because of its many uses, the sweet potato is considered a species of great commercial interest.

The root is a valuable food for human consumption because it is considered to be a good source of energy, minerals, and vitamins. The branches and roots can also be fed to cattle, pigs, poultry, and other domestic animals. The roots of this vegetable crop also have great potential for the production of biomass in biofuel production [1, 2].

Due to the enormous commercial interest in the sweet potato, the conservation of the germplasm of this species is needed. The diversity contained in a germplasm must be protected against losses; the basis of all genetic improvement lies in genetic diversity, which is reflected in the creation of plants with resistance/tolerance to various biotic and abiotic factors, ensuring increased productivity.

*In vitro* conservation frees plants from the elements and risks that occur in the field, reduces costs, ensures the maintenance of genetic fidelity, and facilitates the exchange of germplasm. It is worth noting that, although it is a rugged plant, the sweet potato is susceptible to a large number of diseases caused by fungi, viruses, and nematodes and is also vulnerable to pests such as insects and mites; thus, the Active Germplasm Bank in the field is vulnerable to loss and therefore requires *in vitro* conservation.

The slow growth *in vitro* conservation technique relies on cultures collected in the laboratory using tissue culture techniques. The purpose of this practice is to maximize the period of subculture or extend it indefinitely. To achieve this aim, changes are made in the cultivation environment to slow down or completely suppress the growth of cells and tissues.

Therefore, the aim of this study was to develop a protocol for the *in vitro* conservation of sweet potato [*Ipomoea batatas* (L.) Lam] genotypes.

## 2. Materials and Methods

Stecklings were produced from stem cuttings and planted in plastic pots containing a mixture of sand + cattle manure at a ratio of 2 : 1 and grown in a greenhouse with 50% shade screen, irrigation, and intermittent nebulization located IN the Department of Agronomical Engineering of the Federal University of Sergipe, Brazil.

The trials were conducted at the Laboratory of Tissue Culture and Plant Breeding of the Department of Agronomical Engineering. We used MS culture medium [3] supplemented with 0.7% agar, adjusted to pH 5.8 ± 0.1 followed by autoclave sterilization (121 ± 1°C and 1.05 atm for 15 minutes).

The plant material was kept in a growth room with a photoperiod of 12 hours light and luminous intensity of 60 *μ*mol·m^−2^·s^−1^ via cool white fluorescent light.

The apical segments of the young plants grown in the previous step were used as an explant source for establishing sweet potatoes *in vitro*. Before inoculation, the apical segments were subjected to a two-step disinfestation process. In the first step, the plant material was immersed in tap water for 30 minutes, followed by immersion in Cercobin (4%) for 20 minutes. The second step occurred in a laminar flow chamber, where the segments were immersed in 70% ethanol for 60 seconds, agitated in a solution of 0.5% mercuric chloride for five minutes, and then washed three times in distilled and autoclaved water.

The plant material was cut into segments of approximately 2 cm. The segments were placed in 250 mL flasks containing 25 mL of semisolid MS medium containing sucrose (30 g·L^−1^) and agar (7 g·L^−1^). After inoculation, the material was kept in a growth room with a controlled temperature of 25 ± 2°C. After 30 days of culture, the explants were subcultured onto MS medium to serve as a source of explants for the next steps in the study.

### 2.1. Effect of Different Temperatures and Different Concentrations of Abscisic Acid on Sweet Potato Genotypes

The explants used in this experiment were apical segments of plants already established *in vitro*. The explants were inoculated in test tubes containing 15 mL of culture medium supplemented with varied trial treatments.

We used a completely randomized design, in a 4 × 5 × 2 factorial scheme with five replications, where each replication consisted of five test tubes that each contained a nodal segment. We tested four genotypes (IPB-007, IPB-052, IPB, and IPB-072-137), five concentrations of abscisic acid (0.0, 1.0, 2.0, 4.0, and 8.0 mg·L^−1^), and two temperatures (18 and 25°C).

Every three months, we quantified the survival (%) as well as the height of the shoots using the following rating scale: 1 ≥ 0 and ≤1 cm; 2 ≥ 1 cm and ≤2 cm; 3 ≥ 2 cm and ≤3 cm; 4 ≥ 3 cm and ≤4 cm, 5 ≥ 4 cm and ≤5 cm; and 6 ≥ 5 cm. The color of the shoots was assessed with the following rating scale: 1 = completely green leaves; 2 = start of desiccation and death of the leaves; 3 = between 30 and 50% of dead leaves and shoots; 4 = more than 50% of leaves dead; 5 = leaves and shoots completely dead (adapted from [4]).

### 2.2. Effect of Osmotic Regulator and Salt Concentrations in the MS Culture Medium on Sweet Potato Genotypes

We used a completely randomized design, in a 4 × 3 × 3 factorial scheme with six replications, where each replication consisted of five test tubes that each contained a nodal segment. We tested four genotypes (IPB-007, IPB-052, IPB-072, and IPB-137), three concentrations of MS salts (50, 75, and 100%), and three concentrations of sucrose (10, 20, and 30 g·L^−1^).

Every three months, we evaluated the survival (%), shoot height, and color of the shoots (from the rating scales used in Experiment 1).

### 2.3. Statistical Analysis

All of the data were subjected to analysis of variance with the *F* test and, when significant, the means were compared either by a Tukey test at 5% probability or polynomial regression.

## 3. Results and Discussion

### 3.1. Effect of Different Temperatures and Different Concentrations of Abscisic Acid on Sweet Potato Genotypes

Genotype IPB-007 showed a higher survival rate at 18°C (56%). At 25°C, however, there was not a significant difference between genotypes, with survival rates between 58.40% (IPB-007) and 64.80% (IPB-137) (Table 1). Comparing the two temperatures within each genotype, we found no significant difference between temperatures for genotype IPB-007, which showed survival rates between 56 and 58%. However, the IPB-137 genotype showed a higher survival rate at 25°C (64.80%).

The effect of different ABA concentrations on the IPB-007 genotype is represented by a cubic equation, with the highest average survival obtained with 2.0 mg·L^−1^ ABA. For genotype IPB-137, the different concentrations of ABA are represented by a linear decreasing equation, where the highest average survival rate was obtained at the lowest concentration of the regulator (0.0 mg·L^−1^—78%).

Analyzing the ABA treatment concentrations at 18°C, the variations in shoot height can be demonstrated by a cubic equation, where the lowest average value was obtained at the highest concentration of the regulator (8.0 mg·L^−1^) in genotype IPB-007 (Table 2). Even at 18°C, genotype IPB-137 can be represented by a quadratic equation, with the lowest score (0.33) achieved for shoot height when an ABA concentration of 5.98 mg·L^−1^ was used (Table 2). At a temperature of 25°C, the two genotypes follow a negative linear behavior, producing smaller shoot heights with 8.0 mg·L^−1^ (Table 2).

The different temperatures combined with each concentration of ABA revealed no significant differences in the shoot height variable, with the exception of two genotypes in the absence of the regulator and genotype IPB-007 at a concentration of 1.0 mg·L^−1^ of ABA, which produced a lower mean at a temperature of 18°C.

Independently of temperature, only two conditions showed a significant difference between genotypes at the different concentrations of ABA: in the absence of ABA, genotype IPB-137 (2.02) had a lower mean, and, under a concentration of 1.0 mg·L^−1^ at 25°C, genotype IPB-137 (1.65) had lower shoot heights compared to genotype IPB-007 (3.80).

Regarding ABA within each genotype, the results are represented by a cubic equation where the lowest coloration was obtained with 1.0 mg·L^−1^ (IPB-007) and 4.0 mg·L^−1^ (IPB-137) (Table 3). There was no significant difference between genotypes IPB and IPB-007-137 at each concentration of ABA tested, with the exception of treatment in the absence of ABA, where the IPB-137 genotype had a lower mean score for shoot coloration (Table 3).

Analyzing the effect of ABA within each temperature under 18°C revealed that the concentrations of the regulator did not result in any significant effects on shoot coloration. The 25°C condition was represented by a cubic equation, with the lowest average (2.65) obtained when we used 4.0 mg·L^−1^ of ABA.

At all concentrations of ABA, we observed the lowest means for shoot coloration under 18°C. There are several reports in the literature highlighting the impact of low temperatures on the *in vitro* conservation of different plants: *Piper aduncum* and *Piper hispidinervum* [5], vetiver [6], sugarcane [4], grapevine [7], and *Drosophyllum lusitanicum* [8].

Because it blocks the action of auxin and gibberellins [9] and acts as a hormone to inhibit cell division and elongation [10], ABA is widely used in the *in vitro* conservation of species. Sweet potato plants could be kept under slow growth for a period of 365 days with 10.0 mg·L^−1^ of ABA at 28°C. It should be noted that the regulator inhibited the development of axillary buds without affecting the viability and survival of the explants [11]. In this report, abscisic acid also inhibited the growth of sprouts at the microscale compared to treatment in the absence of the regulator. However, this regulator negatively affects the survival and recovery growth of explants. These different morphogenic responses during *in vitro* sweet potato conservation may be related to the genetic characteristics of each plant because different genotypes were used in both assays. The results regarding the differential behavior of genotypes were reported in previous *in vitro* conservation work of passion fruit, bromeliad, cassava, and others [9, 12, 13].

As demonstrated by the data transcripts, the responses of the different genotypes to the addition of ABA to the culture medium and different temperatures are as diverse as possible. *In vitro* conservation of the green dwarf shows no significant differences between ABA concentrations (0.0, 1.0, 2.0, and 3.0 mg·L^−1^) for any traits at 125 days [14]. The apple can be stored for 21 months with 0.5 mg·L^−1^ ABA and 25% nitrate concentration [15].

In choosing the best treatment for *in vitro* conservation, all of the variables and their effects on genotype conservation should be considered together. Therefore, the sweet potato genotypes IPB and IPB-007-137 could be conserved for 180 days in 2.0 mg·L^−1^ ABA, with a median survival of 78%, and a 68% survival rate was observed for genotype IPB-007 in the absence of ABA at 18°C. With these treatments, it was possible to reduce plant growth and maintain green leaves, allowing the subsequent regeneration and multiplication of the genotypes.

### 3.2. Effect of Osmotic Regulator and Salt Concentrations in the MS Culture Medium on Sweet Potato Genotypes

Regarding MS salt concentrations, we noted that a 50% concentration of the MS culture medium provided a greater survival rate for microplants of genotypes IPB-007 (94.44%) and IPB-052 (88.9%) (Table 4). For genotype IPB-072, the highest survival rates were achieved at 50% (68.89%) and 75% (54.44%) (Table 4). There were no significant differences among treatments for genotype IPB-137. The comparison of the genotypes within each concentration of MS salts indicated that genotypes IPB-007 and IPB-052 had higher survival rates, which do not differ from genotype IPB-137, at the 75 and 50% MS salt treatment level.

At 180 days, there was no significant difference between genotypes IPB-007 and IPB-052 in terms of the sucrose concentrations tested. For genotype IPB-072, a better survival rate was achieved with 20 g·L^−1^ (58.89%) of sucrose (Table 4), but this effect did not differ significantly from the mean obtained with 30 g·L^−1^ of sucrose. For the IPB-137 genotype, higher survival was observed in the 30 g·L^−1^ sucrose treatment. Genotypes IPB-007 and IPB-072 showed higher survival rates in all sucrose concentrations tested (Table 4).

Similar results were obtained for *Helichrysum bracteatum*, whose highest percentages of survival occurred in the 50% MS salts medium supplemented with 15 and 30 g·L^−1^ of sucrose at 18°C [16].

There was no significant difference between the MS salt concentration variations in genotypes IPB-052 and IPB-137 in terms of shoot coloration (Table 5). The genotype IPB-007 showed less shoot coloration with 50 and 100% of MS salts (Table 5). For the genotype IPB-072, the MS salt concentration of 50% resulted in the lowest mean. By comparing the genotypes within each variation of MS salt concentration, it can be seen that IPB-007 obtained the lowest mean shoot coloration when compared to the other genotypes; however, this genotype did not differ from the following treatment/genotype combinations: IPB-052 at the 50% and 75% MS salt concentrations, IPB-072 at the 50% concentration, and 75% MS salts for genotype IPB-137 (Table 5).

At 270 days of storage *in vitro*, we examined survival as a variable in statistical analysis, with double interactions between genotypes × MS salts and sucrose × genotypes (Table 6).

Regarding the concentration of MS salts, we found that higher survival was obtained in genotype IPB-007 (74.44%) with 50% MS salts. For genotype IPB-137, no significant difference between treatments was observed, with survival rates ranging between 47.78 and 53.33%. Comparing the genotypes within each concentration of MS salts, it was observed that IPB-007 showed overall higher survival rates that were indistinguishable from IPB-137 only at the 75% MS salts treatment level. After the same period of *in vitro* conservation, vetiver microplants showed no differences between treatments containing 50% and 75% MS salts, and at a concentration of 100% MS salts, total mortality of the explants was observed [6].

There was no significant difference in the survival of genotype IPB-007 at 270 days between the sucrose concentrations, with rates varying between 60.00 and 65.56%. For the genotype IPB-137 (66.67%), a greater survival rate was achieved at higher concentrations of sucrose (30 g·L^−1^). Comparing the genotypes within each sucrose concentration, it was observed that IPB-007 showed higher survival rates that differed from IPB-137 at all sucrose levels except the treatment with 30 g·L^−1^.

In terms of shoot height, there was an interaction between MS salt concentration × sucrose (Table 7). Comparing the concentration of MS salts within each concentration of sucrose, we found that there was no significant difference among the variations of MS salts when tested at concentrations of 20 and 30 mg·L^−1^ of sucrose. With 10 mg·L^−1^ of sucrose, the 100% MS salt concentration resulted in shoots with lower heights.

At 270 days of cultivation, there was no significant interaction between the sources of variation tested for shoot coloration (Table 8). Genotype IPB-007 (2.65) had a lower mean shoot coloration compared to genotype IPB-137 (2.97). In terms of the different sucrose concentrations we tested, the lowest score was obtained with 30 g·L^−1^ sucrose.

Reducing the levels of MS salts is one of the most popular strategies for the *in vitro* conservation of plants. The strategy is to reduce the amount of nutrients available to the plant, thereby decreasing its growth. For best results, this technique should be combined with other methods, such as lowering the temperature to reduce the metabolism of the plant and adding osmotic regulators to reduce the osmotic potential of the medium, which in turn reduces the absorption of nutrients by the plant. Variations in the MS salt concentration have been tested in several species, including bromeliad [9], cardamom [17], sweet potato [18], and vetiver [6].

Variations in sucrose concentration have been successfully tested for the *in vitro* conservation of various species, such as potato [19], *Drosophyllum lusitanicum* [8], cardamom [17], apple [15], sweet potato [18], cassava [20], and vetiver [21].

In the literature, there are reports of the *in vitro* conservation of *Ipomoea batatas* with variations in MS salts and sucrose. Reference [18] tested four concentrations of MS salts (0, 10, 50, and 100%), four concentrations of sucrose (0, 10, 20, and 30 g·L^−1^), and two temperatures (18 ± 2°C and 27 ± 2°C). At 240 days, sweet potato microplants could be conserved using 100% MS salts and 20 g·L^−1^ of sucrose at a temperature of 18°C.

In this work, the genotypes IPB-007 and IPB-137 could be kept for nine months with an average survival rate of 74.44% and 66.67%, respectively.

## 4. Conclusions

Here we show the successful *in vitro* conservation of sweet potato genotypes IPB-052 and IPB-072 for 180 days, and IPB-007 and IPB-137 for 270 days, both obtained using 30 g·L^−1^ sucrose and 50% MS salts at 18°C.

## Figures and Tables

**Table 1:** Survival of sweet potato plant shoots after 180 days of *in vitro* conservation as a function of abscisic acid concentration, temperature, and genotype.

	Genotype	
	IPB-007	IPB-137
Temperature (°C)		
18	56.00^aA^	31.20^bB^
25	58.40^aA^	64.80^aA^

ABA (mg·L^−1^)		

0,0	64.00^A^	78.00^A^
1,0	58.00^A^	58.00^A^
2,0	78.00^A^	44.00^B^
4,0	42.00^A^	42.00^A^
8,0	44.00^A^	18.00^B^

Equation (*Y*) =	60.115 + 17.116*X* − 8.104*X* ^2^ + 0.714*X* ^3^ *R* ^2^ = 69.56	67.650 − 6.550*X* *R* ^2^ = 87.91

CV (%)	34.85	

The means followed by the same lowercase letters in the same column and uppercase letters in the same line do not significantly differ according to a Tukey test at a 5% probability.

**Table 2:** Height of sweet potato plant shoots after 180 days of *in vitro* conservation as a function of abscisic acid concentration, temperature, and genotype.

ABA	Genotype	
(mg·L^−1^)	IPB-007	IPB-137
	Temperature 18°C	
0	3.83^bA^	2.02^bB^
1.0	1.13^bA^	0.85^aA^
2.0	1.10^aA^	1.10^aA^
4.0	1.40^aA^	0.60^aA^
8.0	0.90^aA^	0.60^aA^

Equation (*Y*) =	4.637 − 3.983*X* + 1.156*X* ^2^ − 0.089*X* ^3^ *R* ^2^ = 93.55	1.765 − 0.490*X* + 0.043*X* ^2^ *R* ^2^ = 77.29

	Temperature 25°C	
0	6.00^aA^	6.00^aA^
1.0	3.80^aA^	1.65^aB^
2.0	1.93^aA^	1.40^aA^
4.0	0.90^aA^	1.03^aA^
8.0	0.40^aA^	0.70^aA^

Equation (*Y*) =	4.452 − 0.616*X* *R* ^2^ = 71.62	3.588 − 0.048*X* *R* ^2^ = 65.45

CV (%)	34.92	

The means followed by the same uppercase letters in the same line and lowercase letters between temperatures do not significantly differ according to a Tukey test at a 5% probability.

**Table 3:** Coloration of sweet potato plant shoots after 180 days of *in vitro* conservation as a function of abscisic acid concentration, temperature, and genotype.

ABA	Genotype	
(mg·L^−1^)	IPB-007	IPB-137
0	3.20^A^	2.53^B^
1.0	2.24^A^	2.83^A^
2.0	2.84^A^	3.09^A^
4.0	2.25^A^	2.35^A^
8.0	3.15^A^	2.75^A^

Equation (*Y*) =	3.059 − 0.455*X* + 0.080*X* ^2^ + 0.003*X* ^3^ *R* ^2^ = 57.24	2.490 − 0.733*X* − 0.293*X* ^2^ + 0.026*X* ^3^ *R* ^2^ = 91.43

	Temperature (°C)	
	18	25

0	2.25^B^	3.48^A^
1.0	2.00^B^	3.07^A^
2.0	2.24^B^	3.69^A^
4.0	1.95^B^	2.65^A^
6.0	1.80^B^	4.10^A^

Equation (*Y*) =	ns	3.346 + 0.320*X* − 0.207*X* ^2^ + 0.022*X* ^3^ *R* ^2^ = 74.45

CV (%)	26.25	

The means followed by the same uppercase letters in the same lines do not significantly differ according to a Tukey test at a 5% probability.

**Table 4:** Survival of sweet potato microplants at 180 days of *in vitro* conservation under 18°C compared between genotypes and the concentration of MS salts and sucrose.

	Genotypes			
	IPB-007	IPB-052	IPB-072	IPB-137
Concentration of MS (%)				
50	94.44^aA^	88.89^aA^	68.89^aB^	64.44^aB^
75	72.22^bA^	77.78^bA^	54.44^aB^	65.56^aAB^
100	68.89^bA^	70.00^bA^	32.22^bB^	61.11^aA^

Sucrose (g·L^−1^)				
10	77.78^aA^	80.00^aA^	41.11^bB^	53.33^bB^
20	74.44^aAB^	81.11^aA^	58.89^aB^	60.00^bB^
30	83.33^aA^	75.56^aA^	55.55^abB^	77.78^aA^

CV (%)	27.70			

The means followed by the same uppercase letters in the same line and lowercase letters in the columns do not significantly differ according to a Tukey test at a 5% probability.

**Table 5:** Coloration of sweet potato plant shoots after 180 days of *in vitro* conservation under 18°C, depending on the genotype and variations in the concentration of MS salts and sucrose.

	Genotypes			
	IPB-007	IPB-052	IPB-072	IPB-137
Concentration of MS (%)				
50	1.16^bB^	1.49^aB^	1.72^bAB^	2.41^aA^
75	1.81^aB^	2.04^aB^	3.07^aA^	2.30^aAB^
100	1.07^bC^	1.94^aB^	3.47^aA^	2.34^aB^

Sucrose (g·L^−1^)				
10	1.06^aC^	1.79^aB^	3.36^aA^	2.64^aA^
20	1.51^aB^	1.84^aAB^	2.31^bA^	2.57^abA^
30	1.57^aB^	1.84^aAB^	2.59^bA^	1.85^bAB^

CV (%)	44.25			

The means followed by the same uppercase letters in the same line and lowercase letters in the columns do not significantly differ according to a Tukey test at a 5% probability.

**Table 6:** Survival of sweet potato plants at 270 days of *in vitro* conservation under 18°C, depending on genotype and the variations of MS salts and sucrose concentration.

	Genotypes	
	IPB-007	IPB-137
Concentration of MS (%)		
50	74.44^aA^	51.11^aB^
75	53.33^bA^	53.33^aA^
100	60.00^bA^	47.78^aB^

Sucrose (g·L^−1^)		
10	60.00^aA^	41.11^bB^
20	62.22^aA^	44.44^bB^
30	65.56^aA^	66.67^aA^

CV (%)	27.34	

The means followed by the same uppercase letters in the same line and lowercase letters in the same column do not significantly differ according to a Tukey test at a 5% probability.

**Table 7:** Height of sweet potato plant shoots at 270 days of *in vitro* conservation under 18°C, depending on genotype and variations in the salt MS medium and sucrose.

Concentration of MS (%)	Sucrose (mg·L^−1^)		
	10	20	30
50	3.15^aA^	3.07^aA^	3.07^aA^
75	3.31^aA^	3.11^aA^	2.82^aA^
100	1.93^bB^	2.56^aAB^	3.26^aA^

CV (%)	27.94		

The means followed by the same lowercase letters in the same column and uppercase letters in the same line do not significantly differ according to a Tukey test at a 5% probability.

**Table 8:** Coloration of sweet potato plant shoots at 270 days of *in vitro* conservation under 18°C as a function of genotype and changes in MS salts and sucrose concentration.

Genotype	Color of shoots
IPB-007	2.65^b^
IPB-137	2.97^a^

Sucrose (g·L^−1^)	

10	3.01^a^
20	3.12^a^
30	2.30^b^

CV (%)	28.28

The means followed by the same lowercase letters in the same columns do not significantly differ according to a Tukey test at a 5% probability.

## References

[1] Monteiro AB (2007). Silagens de cultivares e clones de batata-doce para alimentação animal visando sustentabilidade da produção agrícola familiar. *Revista Brasileira de Agroecologia*.

[2] de Oliveira MKT, Bezerra Neto F, Câmara FA, Dombroski JLD, de Freitas RMO (2008). Multiplicação *in vitro* de batata-doce (*Ipomoea batatas* Lam). *Revista Caatinga*.

[3] Murashige T, Skoog FA (1962). Revised medium for rapid growth and bioassays with tobacco tissue cultures. *Physiologiae Plantarum*.

[4] de Lemos EEP, Ferreira MdS, de Alencar LMC, Neto CER, de Albuquerque MM (2002). Conservação *in vitro* de germoplasma de cana-de-açúcar. *Pesquisa Agropecuária Brasileira*.

[5] da Silva TL, Scherwinski-Pereira JE (2011). *In vitro* conservation of *Piper aduncum* and *Piper hispidinervum* under slow-growth conditions. *Pesquisa Agropecuaria Brasileira*.

[6] Santos TC, Arrigoni-Blank MF, Blank AF, Menezes MMLdA (2012). Conservação *in vitro* de vetiver. *Bioscience Journal*.

[7] Silva RdC, Luis ZG, Scherwinski-Pereira JE (2012). Short-term storage *in vitro* and large-scale propagation of grapevine genotypes. *Pesquisa Agropecuária Brasileira*.

[8] Gonçalves S, Romano A (2007). *In vitro* minimum growth for conservation of *Drosophyllum lusitanicum*. *Biologia Plantarum*.

[9] Moreira MJS (2008). *Conservação in vitro de bromeliáceas [M.S. thesis]*.

[10] Kerbauy GB (2004). *Fisiologia Vegetal*.

[11] Jarret RL, Gawel N (1991). Abscisic acid-induced growth inhibition of sweet potato (*Ipomoea batatas* L.) in vitro. *Plant Cell, Tissue and Organ Culture*.

[12] Faria GA, Costa MAPDC, Junghans TG, Ledo CADS, Souza ADS (2006). Sucrose and sorbitol effect in the *in vitro* conservation of *Passiflora giberti* N. E. Brown. *Revista Brasileira de Fruticultura*.

[13] Macia RJ (2012). *Conservação in vitro de cultivares de mandioca (Manihot esculenta Crantz) [M.S. thesis]*.

[14] Lédo AS, Freire KCS, Machado CdA, de Oliveira LFM, Barbosa SBSC Efeito do ácido abscísico na conservação *in vitro* de coqueiro anão verde do Brasil de Jiqui.

[15] Kovalchuk I, Lyudvikova Y, Volgina M, Reed BM (2009). Medium, container and genotype all influence *in vitro* cold storage of apple germplasm. *Plant Cell, Tissue and Organ Culture*.

[16] Lima-Brito A, Albuquerque MMS, Alvim BFM, Resende SV, Bellintani MC, de Santana JRF (2011). Osmotic agents and temperature on *in vitro* conservation of sempre-viva. *Ciencia Rural*.

[17] Tyagi RK, Goswami R, Sanayaima R, Singh R, Tandon R, Agrawal A (2009). Micropropagation and slow growth conservation of cardamom (*Elettaria cardamomum* Maton). *In Vitro Cellular and Developmental Biology*.

[18] Vettorazzi RG, Carvalho VS, Rodrigues R, Sudré CP, Gravina GdA, Lucas EdF Conservação de batata-doce (*Ipomoea batatas*) por cultivo mínimo *in vitro*.

[19] Gopal J, Chamail A, Sarkar D (2004). *In vitro* production of microtubers for conservation of potato germplasm: effect of genotype, abscisic acid, and sucrose. *In Vitro Cellular and Developmental Biology*.

[20] Londe LN, Alves KA, Ribeiro EB (2012). Efeito de concentrações de sacarose e de meio de cultura sobre a taxa de crescimento de Mandioca variedade BGM, 0116 conservadas *in vitro*. *Revista Trópica*.

[21] Santos TC, Arrigoni-Blank MF, Blank AF (2012). Propagação e conservação *in vitro* de vetiver. *Horticultura Brasileira*.

